# Large-Scale Neural Recording Technologies for Investigating Circuit Dysfunction and Functional Biomarker Discovery in Animal Models of Alzheimer’s Disease

**DOI:** 10.3390/ijms27146444

**Published:** 2026-07-20

**Authors:** Dechuan Sun, Song Wang, Qi Meng, Xinyi Wang, Mona Amiri, Ranjith R. Unnithan, Chris French

**Affiliations:** 1 Neural Dynamics Laboratory, Department of Medicine, The University of Melbourne, Melbourne, VIC 3010, Australia; 2 Department of Electrical and Electronic Engineering, The University of Melbourne, Melbourne, VIC 3010, Australia

**Keywords:** Alzheimer’s disease, miniscope, voltage imaging, Neuropixels, CMOS MEAs

## Abstract

Alzheimer’s disease (AD) is a progressive neurodegenerative disorder associated with amyloid beta accumulation, tau pathology, and neuronal loss. Increasing evidence suggests that AD is associated with disruptions in large-scale circuit activity across multiple disease animal models, even before clear cognitive decline. These findings highlight the need for functional biomarkers that capture dynamic changes in neuronal network activity, rather than relying solely on molecular or anatomical measures. Recent advances in neural recording technologies now allow AD-related network dysfunction to be examined with greater spatial and temporal resolution. This review summarises recent progress in large-scale neural recording technologies, including miniaturized fluorescence microscopy, voltage-sensitive optical recording, high-density probe electrophysiology, and high-density CMOS microelectrode array systems. These techniques provide improved characterization of large network-level abnormalities in AD animal models. Although they are unlikely to serve directly as routine clinical biomarkers, their translational value lies in clarifying circuit-level disease mechanisms, identifying functional features that may inform candidate clinical biomarkers, and supporting preclinical evaluation of therapeutic interventions.

## 1. Introduction

**Figure 1:**
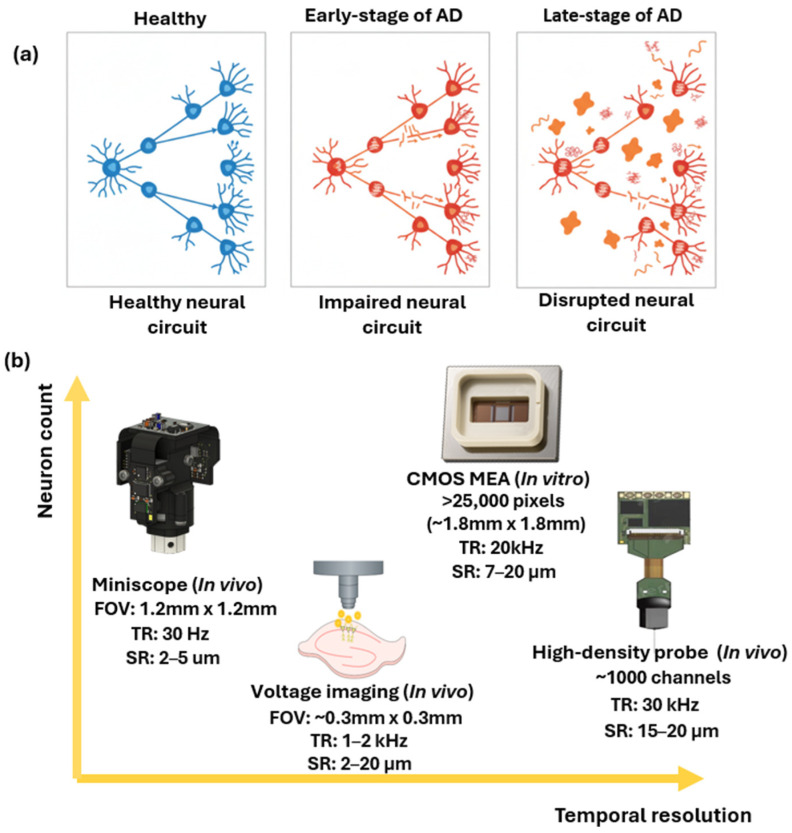
Large-scale neural recording technologies can identify candidate functional readouts of AD-related circuit dysfunction in preclinical models. (**a**) AD exhibits early functional neuronal network impairment associated with disrupted neuronal communication, followed by progressive accumulation of Aβ or tau and eventual neuronal loss at later stages [3,4]. (**b**) Large-scale neural recording technologies, including miniature fluorescence microscopes (miniscopes), high-density probes, optical voltage imaging, and CMOS-based multielectrode arrays (MEAs), have been increasingly applied in AD research. Field of view (FOV), temporal resolution (TR), and spatial resolution (SR) are shown for each technology.

Alzheimer’s disease is a progressive, irreversible neurodegenerative disorder that involves widespread degeneration of the cerebral cortex and hippocampus [1]. Clinically, the disease is characterised by the presence of amyloid beta (Aβ) plaques and neurofibrillary tau tangles within the brain, which form the basis of current diagnostic criteria and disease staging [1,2]. These pathological hallmarks are closely associated with synaptic dysfunction, neuronal degeneration, and progressive cognitive decline [3]. In addition to these pathophysiological biomarkers, recent studies have revealed that alterations in neuronal function and neural network dynamics occur at an early stage of disease development (Figure 1a). Functional abnormalities at the neural network scale have been reported before overt Aβ deposition and tau pathology in animal models, potentially representing early manifestations of disease progression [3,4]. Thus, investigating large-scale neuronal network dynamics in animal models may help identify potential functional biomarkers of AD-related circuit dysfunction, such as measurable network-level features that reflect changes in neuronal communication, synchronisation and population activity patterns. These biomarkers identified in animal models may subsequently guide the development of clinically translatable measures for detecting and monitoring functional changes associated with disease progression. They may also serve as functional endpoints for evaluating therapeutic development.

Several AD animal models have been widely used to investigate the mechanisms underlying these observed abnormalities, such as 5xFAD, APP/PS1, 3xTg-AD, TgF344-AD, and rTg4510 models [5,6]. The 5xFAD and APP/PS1 mouse lines model amyloid-driven pathology through mutations in the human APP and PSEN1 genes, producing robust Aβ plaque deposition in the cortex and hippocampus. However, this pathology emerges earlier and progresses more rapidly in 5xFAD mice. In contrast, 3xTg-AD and TgF344-AD models incorporate tau pathology in addition to amyloid pathology, enabling investigation of the interactions between Aβ accumulation and tau aggregation. However, tau pathology arises differently in the two models. The 3xTg-AD model carries mutant human APP, PSEN1, and MAPT transgenes, whereas the TgF344-AD model expresses mutant human APP and PSEN1 transgenes. In the TgF344-AD model, tau pathology appears later than Aβ pathology. The rTg4510 model provides a complementary tauopathy model, with inducible expression of human MAPT carrying the P301L mutation. This model develops extensive tau tangles, making it particularly useful for studying tau-mediated neurodegeneration. Studies using these models have shown that Aβ and tau associated pathologies alter neuronal excitability, disrupt network synchronisation, and impair normal neuronal firing patterns [7,8]. Notably, these functional alterations are spatially heterogeneous across neural circuits [9,10]. Nevertheless, conflicting findings have also been reported (see Palop et al., 2016 for review) [10], which may in part arise from technical differences in recording methodologies. For example, conventional low-channel multi-electrode array recordings provide limited spatial sampling density, which may fail to capture distributed large-scale network dynamics. In addition, two-photon calcium imaging measures intracellular calcium transients as indirect proxies of neuronal activity and is inherently constrained by slower temporal dynamics compared with extracellular recordings of action potentials, limiting its ability to resolve fast electrophysiological events. Moreover, two-photon imaging is typically performed in head-fixed animals, which may alter naturalistic behavioural states and network dynamics, thereby influencing the interpretation of circuit-level activity. To overcome these limitations, several advanced recording technologies have been applied in AD studies to investigate large-scale neuronal activity with improved spatial and temporal resolution. High-density probes enable extracellular recordings across hundreds to thousands of channels, allowing simultaneous sampling of neuronal spiking activity across distributed brain regions with precise temporal resolution [11,12]. Optical voltage imaging enables direct measurement of membrane potential dynamics with millisecond precision across neuronal populations [13,14]. The development of miniaturized fluorescence microscopes (miniscopes) has enabled longitudinal calcium imaging of large neuronal ensembles in freely moving animals during natural behaviours [15,16]. Microelectrode arrays (MEAs) using complementary metal-oxide-semiconductor (CMOS) technology integrate more than 25,000 electrodes, enabling ultra-high-density extracellular recordings with much improved signal-to-noise ratios from acute brain slices (Figure 1b) [17,18].

In this paper, we have provided an overview of recent technological advancements in large-scale neural recording and their applications in AD research. In particular, we have reviewed one-photon miniscope calcium imaging in freely behaving animals, optical voltage imaging, electrophysiological recordings using high-density probes, and CMOS-based high-density MEA in vitro systems. These studies enable comprehensive characterization of large-scale neuronal network activity and provide deeper insights into AD-related circuit mechanisms. However, their direct clinical use in AD remains impractical. This is largely because miniscopes, voltage imaging, and high-density probes require invasive implantation or surgical preparation, while CMOS MEAs are primarily used for in vitro studies. In addition, optical imaging approaches depend on viral vectors, genetically encoded indicators, or exogenous dyes. These constraints make the technologies unlikely to become routine clinical assessment tools in the near term. Their translational value therefore lies in their ability to clarify circuit-level disease mechanisms, identify functional changes that may serve as clinical candidate biomarkers, support therapeutic evaluation, and improve the construct validity of animal models.

The application of large-scale neural recording technologies to AD models is still at an early stage. Despite the rapid development of these technologies, only a limited number of studies have applied them to investigate circuit dysfunction in AD, with most published only in recent years. This review therefore takes a focused narrative approach. Miniscopes and high-density probes were included because their miniaturized formats enable neuronal activity to be recorded in freely moving animals. Optical voltage imaging was included because it provides both high temporal and spatial resolution. CMOS MEAs were included because they provide dense, repeatable, and experimentally controlled recordings from cultured neurons and acute brain slices. Within each technology section, studies were first considered according to disease-model category, followed by a synthesis of the main functional findings. The limitations of each method and its potential translational relevance were discussed at last.

## 2. Miniaturized Fluorescence Microscope

Calcium acts as a universal secondary messenger that regulates a wide variety of biological processes. Excess Aβ has been found to disrupt neuronal calcium homeostasis, leading to changes in neuronal excitability, synaptic transmission, and network activity that ultimately manifest as functional impairments [19]. Over the past decade, two-photon fluorescence microscopy has been widely used to study these abnormal dynamics in vivo, despite requiring costly femtosecond lasers and being largely restricted to head-fixed animals [20]. In comparison, one-photon fluorescence imaging is readily miniaturized and cost-effective, providing greater experimental flexibility for behavioural studies. Additionally, the development of advanced fluorescence signal processing methods has significantly improved the reliability of neuronal identification and enhanced decoded signal quality [21,22]. Among all open-source miniaturized fluorescence microscopes (miniscopes), the UCLA miniscope has probably been the most impactful. Remarkably lightweight at just 2.6 g, this technology enables long-term calcium imaging of large neuronal populations in freely moving animals [23] and can be applied in real-time [24], facilitating the identification of biomarkers linking AD pathologies to circuit-level dysfunctions [25].

A key advantage of miniscope imaging in AD research is its ability to longitudinally track functional network biomarkers in freely moving animals across disease progression. This approach enables early detection of impairments in information encoding and behaviour associated network modulation, which often precede overt neurodegeneration and progressively intensify with behavioural decline. Recent advances in miniscope technology have significantly improved the imaging quality by enhancing recording stability, increasing spatial resolution, expanding the field of view, and extending recording duration. For example, Skocek et al. developed a high-speed volumetric imaging approach that supported rapid three-dimensional recording of neuronal populations in freely moving rodents [26]. Building on this, Zong et al. achieved large-scale two-photon calcium imaging in freely moving mice, overcoming prior limitations on head fixation and enabling stable recording from thousands of neurons across extended behavioral sessions [27]. More recently, Guo et al. introduced Miniscope-LFOV, a large-field-of-view miniature microscope that can provide single-cell resolution across a large cortical area under both wired and wire-free configurations [28]. Looking forward, these technological advances are expected to facilitate longitudinal circuit-level monitoring, providing a systems-level bridge between molecular pathology, network dysfunction, and behavioural decline in AD studies.

### 2.1. Amyloid-Dominant Models

To clarify how molecular pathology results in functional network impairment, miniscope imaging has been applied across a range of established AD animal models since 2022. These studies have examined AD pathophysiological effects on neuronal population activity and revealed functional alterations consistent with underlying molecular pathology, including Aβ plaque accumulation and tau-associated dysfunction [4]. In a study using 5xFAD mice, Zhang et al. investigated CA1 neuronal calcium activity during immobility and an object location memory task [29]. 5xFAD mice developed abnormal amyloid plaque by 4 months of age, accompanied by significantly reduced CA1 calcium activity during immobility and impaired spatial encoding during the memory task. Notably, object location memory deficit was not observed during this stage but became apparent by 8 months of age, accompanied by severely disrupted neuronal tuning to object location. These results indicate that early hippocampal circuit dysfunction precedes overt memory impairment, supporting the use of circuit-level activity as a potential early biomarker of AD. Further evidence of amyloid related signalling disruption has been reported in studies of associative learning paradigms. Erofeev et al. examined the effects of amyloid pathology on real-time neuronal signalling during fear conditioning in the 5xFAD mouse model [30]. During fear conditioning, 5xFAD mice exhibited attenuated calcium responses compared with controls, along with reduced synchronisation between the stratum pyramidale and hippocampal alveus, as well as decreased consistency of population-level neuronal activity. Combined dysfunction in these subregions may underlie impaired memory formation in 5xFAD mice [31], highlighting activity alterations in the stratum pyramidale and alveus as potential biomarkers of early hippocampal circuit dysfunction.

Amyloid pathology does not produce uniform effects on neural circuits but instead leads to state-dependent alterations in neural activity. Zhou et al. studied how Aβ accumulation affected hippocampal activity in APP/PS1 mice and found that the same pathological peptide can either enhance or suppress neural activity depending on the behavioural state [32]. In their experiment, hippocampal calcium activity was elevated during active exploration and non-rapid eye movement (NREM) sleep, with concurrent disruptions of theta and gamma oscillations involved in memory processing. In contrast, the activity was suppressed during quiet rest and REM sleep. After the administration of cholinergic enhancer physostigmine, excessive neuronal firing in the exploration state was reduced. However, a large number of neurons demonstrated significantly reduced firing activity, resulting in persistent network dysregulation rather than restoring normal circuit function. Moreover, no significant effects of physostigmine were detected on neuronal firing during the quiet rest state. These observations suggest that the effect of physostigmine on neuronal activity may depend strongly on behavioural and physiological state, with different network conditions showing distinct sensitivity to the same intervention. In APP/PS1 mice, one of the earliest measurable circuit abnormalities is the occurrence of brief interictal epileptic spikes within the hippocampal CA1 area and the medial prefrontal cortex during anaesthesia. As the nucleus reuniens (nRE) provides a major thalamic input coordinating hippocampal–prefrontal activity, Shoob et al. investigated whether modulation of this pathway could restore hippocampal network function [33]. Specifically, the authors applied deep brain stimulation to the nRE while monitoring hippocampal activity using miniscope calcium imaging. Tonic stimulation of the nRE reduced epileptic spikes and prevented further deterioration in functional connectivity between the nRE and CA1 neurons. Additionally, the stimulation alleviated deficits in working memory and short-term synaptic plasticity caused by anaesthesia. These results suggest that tonic stimulation of the nRE may help stabilize hippocampal circuitry and improve cognitive resilience, possibly by restoring the neuronal synchrony required for normal memory processing.

### 2.2. Tauopathy Models

In addition to dysfunction associated with hippocampal circuits, another salient feature of AD is cortical network impairment associated with tau proteins. Parka et al. assessed the effects of the aberrant accumulation of tau protein on visual cortical circuit function before the onset of any significant neurodegeneration [34]. This study involved monitoring neuronal calcium activity and local oscillatory activity in the visual cortex of rTg4510 mice at an early stage of pathology. The mice exhibited reduced behavioural responses to visual stimuli, as well as lower levels of stimuli evoked activity for individual neurons. Pharmacologic modulation of inhibitory signalling was unable to restore these deficits, implying that the dysfunction did not arise from inhibition but rather excitatory circuits. Although spontaneous neuronal firing rates were elevated at rest, population synchronization was impaired during visual stimulation displaying reduced theta oscillations. These observations indicate that early alterations in oscillatory dynamics may serve as functional biomarkers of preclinical tau-related neurodegenerative disease.

### 2.3. Models with Combined Amyloid and Tau Pathology

Another widely used model for studying AD is the 3xTg-AD mouse model, which exhibits age-dependent accumulation of extracellular amyloid deposits and neurofibrillary tangles of tau protein in the hippocampus and neocortex [35]. Lin et al. studied the spatial coding characteristics of hippocampal CA1 neuronal ensemble in age-matched 3xTg-AD mice and control mice [36]. Recordings were performed during open-field exploration and track-based navigation tasks. Both young and aged 3xTg-AD mice showed increased calcium activity in CA1 excitatory neurons compared with controls, consistent with previous reports of hippocampal hyperexcitability in AD models [37]. However, 3xTg-AD mice demonstrated significantly reduced spatial encoding quality, as reflected by lower spatial information scores and decreased spatial coherence, with deficits worsening with age. Additionally, locomotion speed had a stronger influence on calcium activity amplitude in AD mice than in controls. Such locomotion-related abnormalities may arise from weakened inhibitory interneuron regulation and impaired septo-hippocampal theta coordination, leading to excessive amplification of locomotion-driven excitatory input. Similarly, Murano et al. reported that neuronal populations in the dentate gyrus exhibited impaired encoding of navigational variables in α-calcium/calmodulin-dependent protein kinase II heterozygous knockout mice, which displayed spatial memory deficits resembling those observed in 3xTg-AD mice [38].

### 2.4. Pharmacological Models of AD-like Network Dysfunction

Beyond structural and firing rate abnormalities, AD pathology also disrupts higher-order network organization. One emerging framework for characterising such dysfunction is neural criticality. Neural criticality describes a dynamical regime in which brain networks operate near a phase transition between order and disorder, enabling optimal information transmission [39]. In a recent study, we recorded CA1 calcium activity in freely moving mice and analysed hippocampal network dynamics at rest and during a novel object recognition task [40]. During task engagement, network dynamics shifted towards a critical regime, reflecting a balance between overly stable and overly chaotic activity. However, this adaptive tuning was disrupted following administration of scopolamine, a muscarinic antagonist that induces transient memory deficits resembling those observed in AD [41]. Changes in criticality under pharmacological perturbation suggest that neural criticality may serve as a functional biomarker of network dysfunction in AD.

### 2.5. Functional Interpretations of Miniscope Findings

Miniscope studies suggest that AD-related circuit dysfunction is not defined by a uniform increase or decrease in neuronal activity, but by a loss of reliable ensemble organisation. In Aβ-dominant models, amyloid pathology consistently disrupts hippocampal network function, although the direction of activity change differs across studies. In 5xFAD mice, early amyloid accumulation is associated with reduced CA1 calcium activity during immobility and degraded spatial coding [29]. Similarly, during fear conditioning, 5xFAD mice show attenuated hippocampal calcium responses and reduced brain regions synchronisation [30]. These results may indicate that amyloid pathology weakens the coordinated hippocampal signalling required for associative memory formation. However, Aβ-related dysfunction is not always expressed as hypoactivity. In APP/PS1 mice, hippocampal calcium activity is increased during exploration and slow-wave sleep but reduced during quiet wakefulness and REM sleep [32]. These apparently contradictory findings are likely to reflect differences in model-specific pathology, behavioural state, and circuit context. The 5xFAD model develops aggressive amyloid pathology and was examined during immobility or associative learning, whereas APP/PS1 mice show more state-dependent disruption across exploration, quiet wakefulness, REM sleep, and slow-wave sleep. Thus, amyloid pathology may not impose a single excitability phenotype, but instead disrupt the ability of hippocampal circuits to regulate gain across behavioural and oscillatory states. A mechanistically related example comes from APP/PS1 experiments targeting the nRE-CA1 pathway, which is involved in regulating hippocampal excitation-inhibition balance. In this model, epileptiform activity and impaired nRE-CA1 functional connectivity were accompanied by working memory deficits, whereas nRE stimulation partially restored circuit homeostasis and improved memory performance [33]. Tau-dominant pathology produces a related but mechanistically distinct pattern. In rTg4510 mice, miniscope imaging reveals impaired visually evoked cortical ensemble responses with increased spontaneous activity [34]. These results suggest that tau pathology may preferentially compromise stimulus-driven population coordination rather than merely altering baseline firing rates. The distinction between spontaneous activity and task evoked coding is important, because elevated baseline activity may coexist with reduced information transfer when neuronal firing becomes poorly timed or weakly coordinated. This dissociation is also evident in the combined Aβ-tau 3xTg-AD model, where CA1 neurons show increased calcium activity but reduced spatial information and spatial coherence [36]. Therefore, increased activity in AD models should not be interpreted as preserved or enhanced circuit function. It may instead reflect noisy excitation, impaired inhibitory control, or weakened septo-hippocampal regulation that reduces the precision of spatial coding. Together, these studies indicate that raw calcium-event frequency is unlikely to serve as a robust biomarker across AD models. More reproducible miniscope-derived biomarker candidates are likely to include coding precision, behavioural state-dependent activity modulation, and task evoked response fidelity.

## 3. Optical Voltage Imaging

Calcium imaging monitors intracellular calcium fluctuations using fluorescent indicators, but is limited by the relatively slow dynamics of calcium signalling. In contrast, optical voltage imaging provides a more direct and temporally precise measure of neuronal activity. It enables fast measurements of membrane voltage signals across neuronal populations, allowing activity to be captured at the level of extended neural networks [14,42,43,44]. Although conventional MEA recordings can achieve comparable temporal resolutions to voltage imaging, they are less practical for large networks due to relatively low channel numbers and insufficient spatial sampling. Compared with high-density probe recordings, optical voltage imaging provides direct visualisation of membrane-potential dynamics and can enable cell-type specific recording through genetically targeted expression of voltage indicators. By integrating high temporal precision with wide spatial coverage, voltage imaging enables detailed analysis of distributed circuit dynamics. This capability is particularly useful for studying AD, where disrupted signal propagation and impaired synchronisation across large neural networks are key features of circuit dysfunction [45]. Despite these advantages, the technical complexity of experimental procedures and the requirement for highly sensitive, high-speed imaging systems have constrained the widespread adoption of voltage imaging, and its application to AD study remains limited.

### 3.1. Amyloid-Dominant Models

Amyloid pathology can impair neural communication not only by altering local excitability, but also by disrupting the propagation of activity across cortical and axonal networks. In an in vitro study using cortical slices from 5xFAD mice, Patel et al. characterised synaptically evoked signal propagation across cortical networks [46]. Neuronal activity was recorded simultaneously at 1 kHz across multiple cortical sites using a fluorescent voltage indicator expressed in pyramidal neurons, revealing disrupted temporal coordination of signal propagation. In particular, activity spread exhibited frequency-dependent vulnerability during external stimulation, with gamma-band stimulation producing the most pronounced propagation deficits. These findings are consistent with AD pathology selectively disrupting high-frequency cortical signal transmission, that has been associated with cognitive information integration [47]. Beyond amyloid plaque deposition, AD is also associated with plaque-associated axonal spheroids that may impair long-range signal propagation [5]. Yuan et al. used two-photon voltage imaging with the genetically encoded voltage sensor ASAP3 to assess antidromic action potential conduction in 5xFAD mice and WT controls [48]. In this experiment, axons were electrically stimulated, and voltage responses were recorded from neuronal somata to determine whether action potentials successfully propagated back to the cell body. Compared with WT controls, 5xFAD mice showed less reliable somatic voltage responses after axonal stimulation, indicating impaired action potential conduction. The study further showed that a higher stimulation threshold was required to evoke successful propagation in 5xFAD axons, consistent with conduction delay or blockade caused by plaque-associated axonal spheroids. These findings demonstrate that amyloid-associated axonal pathology can compromise long-range electrical signalling and thereby contribute to circuit-level dysfunction in AD.

Complementing these findings in 5xFAD mice, studies in APP/PS1 models suggest that amyloid-related circuit dysfunction also involves abnormalities in hippocampal gating, cortical excitability, and network synchronisation. Hazra et al. used fast voltage-sensitive dye imaging to assess hippocampal network dysfunction in APP/PS1 mice [49]. The experiments were performed in hippocampal slices with intact entorhinal connections derived from mice aged 12 to 16 months with significant amounts of amyloid deposition. The dentate gyrus acts as a gate in a normal brain for controlling excitation in the hippocampus from entorhinal cortex neurons. In the case of APP/PS1 mice, impaired inhibitory regulation led to abnormal DG hyperexcitability, disrupting the precise hippocampal neuronal firing patterns required for memory encoding. This dysfunction may result from a combination of intrinsic DG hyperexcitability and reduced inhibitory control within hippocampal circuits. The dysfunction of circuits in AD has been proposed to follow a biphasic trajectory during disease progression [50]. To characterise network excitability changes across disease stages, Zhu et al. used fast voltage-sensitive dye imaging to examine cortical population membrane potential dynamics in the fronto-parietal cortex of an APP/PS1 mouse model before and after amyloid plaque deposition [51]. Prior to overt plaque accumulation, cortical circuits exhibited increased evoked depolarization amplitude, consistent with early-stage hyperexcitability [52]. In contrast, after plaque accumulation, cortical depolarization dynamics became disrupted, characterized by reduced response reliability and impaired propagation of activity across cortical circuits. By capturing subthreshold membrane potential fluctuations, these data support a biphasic evolution of cortical circuit dysfunction in AD, transitioning from early hyperexcitability to later-stage propagation deficits. Extending these observations, Maatuf et al. examined population-level neuronal responses in the barrel cortex of an APP/PS1 AD mouse model [53]. Cortical circuits exhibited reduced response fidelity, with whisker evoked population activity becoming less consistent across trials. In addition, cortical responses showed abnormal amplitude fluctuations, including sporadic episodes of excessive population-level activation. Notably, these functional abnormalities were observed in the absence of significant neuronal loss. Despite these alterations, stimuli evoked response synchrony remained largely preserved and did not differ significantly from controls. In contrast, spontaneous cortical activity in the AD model exhibited reduced synchrony. Collectively, these findings suggest that amyloid pathology disrupts intrinsic network coordination, leading to dysfunctional cortical dynamics.

### 3.2. Functional Interpretations of Voltage-Imaging Findings

Current voltage-imaging studies in AD have mainly focused on Aβ-dominant models. In 5xFAD mice, voltage imaging has shown that amyloid pathology disrupts fast electrical signalling. Synaptically evoked depolarisations exhibited impaired propagation across cortical circuits, with high-frequency stimulation producing pronounced deficits [46]. At a more structural level, plaque-associated axonal spheroids can impair action-potential conduction, producing conduction delay or blockade between axons and neuronal somata [48]. These findings suggest that Aβ pathology can compromise the temporal coordination required for rapid information transfer across neural networks. In APP/PS1 mice, dentate-gyrus hyperexcitability and weakened inhibitory gating were observed [49]. Consistently, in the barrel cortex of aged APP/PS1 mice, whisker stimulation evoked abnormally large population-level voltage responses, while spontaneous cortical synchrony was reduced [53]. However, the hyperexcitable state is not stable across disease progression [51]. A possible reason for this phenomenon is that early Aβ-related synaptic and inhibitory dysfunction may initially increase network responsiveness. However, as plaque burden, synaptic dysfunction and neuritic damage progress, neural networks may gradually lose the capacity to transmit activity efficiently. Overall, amyloid pathology appears to impair the timing, reliability and propagation of membrane-potential signals across local and long-range networks. These abnormalities may weaken coordinated information transfer between brain regions and contribute to the progressive breakdown of circuit function in AD.

## 4. High-Density Probes

Initial electrophysiological studies of AD relied primarily on relatively low-density electrode arrays, which limited the number of simultaneously recorded neurons and constrained the characterization of distributed network dynamics [54]. To overcome these limitations, two-photon calcium imaging has been introduced to enable population-level recording across a broader field of view. While this approach significantly improves spatial sampling, it is inherently biased toward recording neurons in superficial layer 2/3. Although imaging deeper layers is possible, it typically requires aspiration of overlying tissue, which may disrupt normal circuit architecture [55]. Furthermore, although genetically encoded calcium indicators have undergone substantial improvements in kinetics, sensitivity, and signal stability, their temporal resolution is still insufficient to fully resolve action potential level neural dynamics [56]. These methodological constraints may introduce sampling bias by skewing recordings toward local microcircuit activity while limiting detection of rapid spiking events and distributed network dynamics. With advances in nanofabrication and probe technology, recent large-scale electrophysiological studies using high-density probes have enabled more precise, longitudinal observation of neural circuit activity [57]. Among these technologies, Neuropixels probes have recently gained significant attention in AD research, providing high channel density and recording stability for large-scale in vivo recordings of neural population activity. They also allow distinction between excitatory and inhibitory neurons based on action potential properties. However, the high cost and complexity of analysing data from such a large number of recording channels have limited their widespread application. As a result, studies using high-density probes in AD research have only emerged in recent years.

### 4.1. Amyloid-Dominant Models

High-density probe studies of amyloid-dominant models suggest that early AD-related circuit dysfunction is not globally distributed, but instead emerges in specific cortical layers, cell types, and network states. In APP/PS1 mice, Papanikolaou et al. observed abnormal calcium transients in visual cortex layer 5 neurons before any significant plaque formation, as shown by two-photon imaging [58]. Recording from the same cortical area using a Neuropixels probe demonstrated that the transient events were associated with decreased neural network activity as well as impaired visually evoked responses in layer 5/6 parvalbumin-positive fast-spiking interneurons (PV-FSIs). In contrast, neuronal activity in superficial layers was not affected. In addition to these deficits, there was evidence of a decline in NPTX2 levels in the deeper layers of the cortex, a reduction in GluA4 receptors in PV-FSIs, and a decrease in excitatory inputs to PV-FSIs. Collectively, these findings imply that PV-FSIs represent an early vulnerable target in AD before significant cognitive impairment. These findings may be explained by previous studies by Verret et al. (2012), which used brain slice electrophysiology to demonstrate that dysregulation of Nav1.1 channels impairs the function of PV-FSIs in AD models [59]. Nav1.1 is thought to be the dominant sodium channel subtype in interneurons. Similarly, Klee et al. investigated the neural activity within the frontal cortex in APP/PS1 mice at the early stage of the disease using Neuropixels probes [60]. Specifically, APP/PS1 mice demonstrated reduced spontaneous firing rates of pyramidal neurons accompanied by increased local field potential (LFP) power in theta and beta frequency bands. Additionally, low-firing pyramidal cells showed high phase coupling with ongoing theta and beta rhythms. These findings contrast with the hippocampal hyperactivity commonly observed in AD models [61]. Moreover, acute administration of the antiepileptic drug levetiracetam restored pyramidal neuron firing rates and LFP power towards wild-type levels and reduced pathological coupling between pyramidal cells and interneurons. These results are consistent with clinical reports by Bakker et al., showing the effectiveness of levetiracetam for mild cognitive impairment [62]. Altogether, these data suggest that AD associated microcircuit alterations arise from heterogeneous, region and layer dependent patterns of dysregulated neuronal activity, rather than from uniform global network dysfunction. In line with this, levetiracetam shows promise as a targeted approach to restore aberrant neural activity in AD, particularly by restoring pyramidal neuron firing and network synchrony.

### 4.2. Models with Combined Amyloid and Tau Pathology

It has been hypothesized that dysfunction of cerebral cortical microcircuits constitutes a key component of AD pathogenesis [10,63]. Chen et al. studied neural activity in both the hippocampus and the primary somatosensory cortex of TgF344-AD rats at an early stage of the disease, prior to the onset of cognitive deficits [64]. This animal model shows pathological features of both amyloidosis and tauopathy, and cognitive deficits usually appear by the age of 12 months. Cognitively resilient rats were identified based on their performance in the Barnes maze test. Their neural activity during repeated somatosensory stimulation was subsequently recorded using Neuropixels silicon probes. In total, 8500 neurons were recorded across all animals. Compared to their non-transgenic littermates, cognitively resilient AD rats recruited fewer neurons but exhibited more robust neuronal representations during repeated stimulation. This pattern was evident in both hippocampal inhibitory and cortical excitatory neuronal ensembles. In addition, these rats showed reduced burst-like firing in excitatory neurons during stimuli evoked activity and exhibited altered patterns of functional synaptic connectivity. It is worth noting that population level firing activity remained preserved regardless of the extent of Aβ and tau deposition.

In another model using APP/PS1-rTg4510 mice, Harris et al. performed in vivo patch clamp and Neuropixels recordings at the age of 9 months, a stage in the course of the disease where amyloid and tau pathologies are well established [65]. Compared with 3xTg-AD mice, APP/PS1-rTg4510 mice combine an APP/PS1 amyloid background with high-level mutant tau expression from the rTg4510 line, resulting in a more tau-intensive model of amyloid–tau pathology. The authors showed that tau pathology selectively disrupted burst firing patterns in pyramidal cell networks while largely preserving single spike activity. The selective impairment is consistent with the hypothesis that tau does not inhibit neuronal excitation globally but selectively affects higher order firing patterns that are vital for proper temporal coding and plasticity. Unlike tau pathology, Aβ pathology did not show the same impact on neuronal firing patterns. Instead, burst firing impairments were correlated with alterations in theta oscillations and high frequency ripple events, which were known to be important for learning and memory processes. In addition, there was a reduction in neuronal expression of CaV2.3 voltage-gated calcium channels, which were involved in regulating burst firing activity. Furthermore, a soluble high molecular weight form of tau was identified in brain tissue preparations and demonstrated to suppress burst firing, leading to severely disrupted neuronal temporal coding. These findings suggest a cellular mechanism linking tau pathology to cognitive impairment in AD and identify an intracellular tau subtype as a potential therapeutic target.

### 4.3. APP-Related Mechanisms of Circuit Dysfunction

Beyond classical amyloid- and tau-transgenic models, recent high-density recording studies have begun to examine how APP-related mechanisms in specific cell types contribute to circuit dysfunction in AD. Harris et al. combined two-photon calcium imaging with Neuropixels recordings to investigate neocortical and hippocampal network dynamics in APP knockout mice [66]. APP family proteins are widely expressed in the brain and are closely associated with AD pathology. In these mice, baseline firing rates were reduced across rest, locomotion, and task engagement, particularly in excitatory neurons, with many neurons exhibiting silent or low-activity profiles. At the network level, cortical neurons showed reduced phase locking to hippocampal rhythms and weakened coactivity patterns. In addition, attenuated transitions between rest-like and active states were observed, implying impaired switching between low and high engagement firing regimes. Functionally, APP knock-out reduced the synaptic expression of GluN1 and caused impairment in NMDA receptor function [67]. Consistently, blocking of NMDA receptors in healthy mice induced changes in neural networks as described above, while activation of NMDA receptors in mice lacking APP protein improved neural activity and alleviated certain behavioural impairments. This evidence highlights NMDA receptor dysfunction as an important pathogenic factor in AD.

In addition to neuron-centric mechanisms, glial contributions to AD pathology have also been identified. Rajani et al. demonstrated that Aβ production is not restricted to neurons, challenging the conventional view that neurons are the principal source of pathogenic Aβ [68]. The authors showed that oligodendrocytes, which are primarily known for forming myelin, also produce substantial amounts of Aβ. Using both human tissue and an APP NL-G-F knock-in AD mouse model, they found that Aβ originating from oligodendrocytes contributed to neuronal hyperactivity, a phenotype commonly observed in the early stages of AD. Notably, selective reduction in Aβ production in oligodendrocytes decreased AD-associated neuropathology, restored neuronal activity toward normal patterns, and promoted recovery of dysfunctional neural circuits in vivo. These outcomes demonstrate that oligodendrocyte derived Aβ is not merely a byproduct but a likely significant contributor to neural dysfunction.

### 4.4. Functional Interpretations of High-Density Probe Findings

Together, these results suggest that AD-related network dysfunction is cell type, layer, and state-dependent. In Aβ-dominant models, early cortical dysfunction appears particularly selective. In APP/PS1 mice, deep layer 5/6 PV-FSIs exhibit reduced spiking and impaired visual tuning before substantial plaque formation, whereas superficial PV interneurons remain largely unaffected [58]. However, frontal cortical pyramidal cells exhibited reduced firing and abnormal coupling to ongoing oscillations [60]. These findings may appear inconsistent, but they likely reflect differences in brain region, cell type, disease stage, and network state. Aβ pathology may increase activity in some vulnerable circuits while suppressing firing or impairing tuning in others, particularly when interneuron dysfunction, altered inhibition, and compensatory oscillatory coupling reshape local excitation-inhibition balance. This interpretation is partially supported by evidence from App NL-G-F mice showing that oligodendrocyte-derived Aβ contributes to early neuronal hyperactivity [68]. Selective suppression of oligodendrocyte Aβ production can restore neuronal activity towards normal patterns [68]. In combined Aβ-tau models, preserved population-level firing does not necessarily reflect preserved network function. In cognitively resilient TgF344-AD rats, population-level firing activity remained relatively preserved despite Aβ and tau pathology. Intriguingly, these animals showed more robust stimulus representations but with reduced excitatory burst-like firing patterns [64]. These findings suggest that cognitive resilience may depend on how neural activity is organised, rather than on the preservation of mean firing activity alone. Similarly, APP/PS1-rTg4510 mice showed selective disruption of hippocampal burst firing patterns, while single-spike generation was largely preserved [65]. Together, these studies suggest that AD-related circuit dysfunction is marked by selective impairments in how neuronal populations encode and synchronise. Therefore, measures of coding stability and burst structure may capture circuit dysfunction more sensitively than mean firing rate alone.

## 5. CMOS-Based High-Density Microelectrode Arrays

Microelectrode arrays (MEAs) have been widely used to record spontaneous and evoked activity in cultured neuronal networks, providing a practical and reproducible approach for investigating population level neural dynamics [69]. The main drawback of MEA recordings has been the low density of electrodes, which affects the spatial resolution and leads to biased estimates of neuronal firing rates and synchrony. Crucially, low-density recording configurations may fail to capture spatially confined circuits that can disproportionately influence overall network behaviour. This limitation may partly explain why cultures subjected to the same biological treatment often produce inconsistent results when measured using conventional MEAs [70]. To overcome these limitations, high-density microelectrode arrays (HD-MEAs), particularly those based on CMOS technology, have been developed [71,72]. This technology enables simultaneous recording and stimulation across thousands of densely packed channels and has been validated in vitro. In conventional low-density MEA systems, typically comprising 32 to 60 recording channels, the inter-electrode spacing is often much larger than the characteristic scale of local neural microcircuits. Consequently, each electrode may capture extracellular signals originating from numerous neighbouring neurons, thereby complicating accurate spike sorting.

### 5.1. Aβ-Induced Network Dysfunction

Utilising the improved spatial sampling capability of HD-MEAs, several studies have explored how AD-related pathological factors influence population-level neural communication. Amin et al. investigated Aβ induced changes in neural communication using rat hippocampal neurons cultured on high-density CMOS MEAs with over 4000 recording channels [73]. The study used an in vitro AD drug model by applying synthetic Aβ42 peptides directly to cultured rat neurons. The neural network showed spatially heterogeneous Aβ-induced dysfunction, manifested as clusters of hyperactive neurons interspersed with regions of reduced neuronal activity. This heterogeneous excitability was consistent with in vivo observations showing that Aβ pathology caused localised areas of excessive neuronal activity [74,75,76]. Under control conditions, cultured neuronal networks exhibited well organised bursting activity characterised by stable spatiotemporal patterns, whereas Aβ related perturbations disrupted burst synchronisation, timing, and propagation patterns across the network. Additionally, overstimulation of extrasynaptic NMDA receptors further disrupted network properties and contributed to progressive deterioration of neuronal network function. In contrast, neural stem cell therapy and pharmacological treatments using memantine and saffron extract mitigated Aβ-induced dysfunction and restored physiological network firing patterns. Similar findings were reported in a more recent study by Ganbat et al. on primary cortical neuron cultures [77]. Network connectivity analysis further revealed reduced inter-neuronal connection strength and a substantial loss of highly connected hubs. Global network efficiency was also decreased, indicating impaired information transmission across the network. These studies indicate that Aβ related pathology disrupts neural network function across multiple scales. Such disruptions are likely to alter neuronal excitability, temporal synchronization, and connectivity patterns, ultimately impairing efficient information processing within neural circuits.

### 5.2. Tau-Associated Network Dysfunctions

In addition to Aβ pathology, tau protein aggregation and propagation represent another major mechanism contributing to neurodegeneration in AD [78]. Tau pathology has been associated with network hypoactivity in several studies [78]. However, it remains unclear whether tau-associated hypoactivity represents authentic neuronal suppression. Alternatively, it may be partly influenced by reduced spike detectability during low firing activity and insufficient spatial sampling associated with limited electrode channels. Conventional spike sorting pipelines often show reduced sensitivity to low-amplitude spikes under low-activity conditions, which may lead to underestimation of tau associated hypoactivity [79]. To address this concern, Brockhoff et al. developed a self-supervised spike sorting algorithm with an iterative fine-tuning approach to improve sorting accuracy [80]. Neural activity was recorded from hippocampal neuronal cultures exposed to monomeric tau using a MaxWell HD-MEA system with 26,400 channels [80]. This cellular model was designed to mimic tau-induced neurodegenerative stress observed in AD. The pathological tau exposure impaired neuronal excitability, as reflected by decreased firing frequency and prolonged inter-spike intervals, while spike amplitude was relatively preserved. At the population level, network burst frequency declined and spike events became less synchronized. Additionally, analyses of functional connectivity revealed impaired neuronal coupling, reflected by decreased correlations between units and reduced temporal precision of spike activity across the electrode array. The overall activity level declined progressively over time, accompanied by an increase in the fraction of silent or weakly active units. These findings provide more reliable evidence supporting tau-associated suppression of neuronal activity. In another study using the MaxWell system, Mueller et al. introduced disease-relevant tau seeds into primary neuronal cultures derived from MAPT knock-in mice and reproduced intracellular tau aggregation under controlled conditions [81]. In contrast to the neural network alterations reported by Brockhoff et al., tau seeding in AD models was associated with network hyperexcitability and hypersynchrony. Similarly, the seeding process triggered fast degeneration of the axons before substantial neuronal loss and synapse malfunction occurred. This probably represents an early phase of pathology in which acute tau aggregation disrupts axonal transport and compromises synaptic integrity without causing substantial neuronal death. Such alterations may destabilize excitation-inhibition balance and enhance NMDA receptor dependent network responsiveness, leading to transient hyperexcitability before the transition toward more persistent functional decline.

### 5.3. Functional Interpretations of CMOS MEA Findings

CMOS MEA studies suggest that Aβ and tau disrupt neuronal network function through partially distinct but convergent mechanisms. In Aβ-exposure models, synthetic Aβ42 produces spatially heterogeneous network dysfunction, with hyperactive neuronal clusters coexisting with regions of reduced activity, accompanied by impaired burst timing, disrupted synchronisation, abnormal propagation, weakened functional connectivity, and loss of highly connected network hubs [73,77]. This observation is consistent with the hypothesis that Aβ affects the balance between excitatory and inhibitory neurotransmission, causing local hyperexcitability and reducing the overall efficiency of brain networks [3]. Tau-associated models show an additional source of heterogeneity. Monomeric tau exposure is associated with reduced firing frequency, prolonged inter-spike intervals, decreased burst frequency, and weaker synchronisation [80]. In contrast, tau seeding with AD-relevant tau aggregates can produce early hyperexcitability and hypersynchrony before substantial neuronal loss, together with axonopathy and synaptic dysfunction [81]. These divergent findings are likely to reflect differences in tau species, aggregation state, and exposure duration. Soluble or monomeric tau may directly suppress neuronal excitability or impair synaptic transmission, whereas seeded pathological tau may first destabilise axonal and synaptic compartments, producing transient network hyperexcitability before later functional decline. Together, CMOS MEA studies show that AD-related dysfunction emerges as a breakdown of network organisation in simplified neuronal systems. The utility of such research lies not in the recreation of the full pathology of the actual brain, but rather in the understanding of the ways that Aβ and tau affect burst patterns, and connectivity under controlled experimental conditions.

## 6. Current Limitations and Future Challenges

### 6.1. Miniscope

Miniscopes enable calcium imaging from large neuronal populations in freely moving animals, but their performance remains constrained by motion artefacts, optical access, and the intrinsic kinetics of calcium indicators. Movement-related artefacts can substantially degrade imaging quality, particularly during active behavioural tasks. In rodents, most motion artefacts arise from in-plane translational displacement and can often be corrected using rigid image registration algorithms [82]. However, this assumption may not hold in larger animals, such as sheep, where brain tissue can undergo local deformation. In such cases, non-rigid registration approaches may provide more effective correction by accounting for spatially heterogeneous tissue movement [82]. Out-of-plane motion remains more difficult to correct, because axial displacement changes the focal plane and can cause neurons to move in and out of focus. Volumetric imaging may help address this limitation by capturing activity across multiple depths, although this comes at the cost of increased optical and computational complexity [83].

A further limitation of miniscope calcium imaging is that it provides an indirect readout of neuronal activity. Although recent genetically encoded calcium indicators, such as jGCaMP8f, have markedly improved temporal resolution compared with earlier GCaMP variants, calcium signals remain slower than membrane voltage changes [84]. As a result, miniscope calcium imaging is still limited in its ability to resolve individual action potentials, high-frequency spike trains, and fast temporal interactions between neurons. This temporal constraint is particularly relevant when studying Alzheimer’s disease-related changes in synchrony, oscillatory coupling, and rapid circuit dynamics. In addition, long-duration recordings may be affected by photobleaching and phototoxicity. Noticeable photobleaching can occur after approximately 30–60 min of continuous imaging, although the onset and severity depend on illumination intensity, and indicator expression level. These effects may complicate longitudinal comparisons by introducing gradual signal loss that is unrelated to disease progression.

Optical access also imposes important anatomical constraints. For superficial cortical recordings, miniscope imaging can be performed relatively directly through a cranial window. In contrast, imaging deep brain regions usually requires implantation of a cannula or gradient-index relay lens. When a small field of view is sufficient, a metal cannula containing a relay lens can be implanted above the target region. However, achieving a larger field of view in deep structures may require removal of overlying cortical tissue, so that the relay lens can be positioned directly above the region of interest. This increases surgical invasiveness and may disrupt surrounding circuits, thereby affecting the interpretation of recorded activity. Therefore, although miniscope imaging is highly valuable for longitudinal and behaviourally relevant studies of AD models, its data must be interpreted in light of motion correction limits, slow calcium kinetics, photobleaching, phototoxicity, and the anatomical compromises required for deep-brain imaging.

### 6.2. Voltage Imaging

While voltage imaging provides a more accurate temporal measurement of neuronal electrical dynamics compared to calcium imaging, it also faces some technical issues. Like calcium imaging, voltage imaging can be affected by motion artefacts, photobleaching, phototoxicity, and constraints on optical access. Compared with calcium indicators, voltage indicators generally produce smaller fractional fluorescence changes, making voltage signals more vulnerable to background fluorescence [85]. Therefore, voltage imaging typically requires highly sensitive cameras. This requirement creates several trade-offs between sensitivity, field of view, and recording duration. The large numerical-aperture optical system improves photon collection efficiency and signal-to-noise ratio, but this also restricts the observable field of view. Increasing illumination intensity can partially compensate for weak fluorescence signals, but this accelerates photobleaching and increases the risk of phototoxicity. In addition, voltage imaging requires very high frame rates (>300 FPS) to resolve rapid membrane potential dynamics. The resulting high data throughput imposes significant challenges for sensor readout and data transfer. Consequently, such cameras are usually designed with fewer pixels to achieve the required acquisition speed. Beyond hardware limitations, the development of voltage indicators remains more challenging than that of calcium indicators. Genetically encoded voltage indicators must simultaneously balance brightness, voltage sensitivity, response kinetics, and photostability, and only a limited number of indicators currently provide sufficient performance for large-scale in vivo recordings [13].

### 6.3. High-Density Probes

High-density electrophysiological probes allow for accurate recording of spike trains at millisecond timescale in hundreds to thousands of channels. Their depth-resolved recordings further enable the investigation of distributed neural activity. Due to recent advances in fabrication technologies, these probes achieve smaller implant footprints and therefore cause less tissue damage than optical approaches. However, long-term recording stability remains a major challenge, as signal quality often declines over weeks to months due to tissue reactions and probe movement [86]. These factors may affect spike amplitude, waveform shape, and unit detectability. Thus, it may be difficult to identify whether observed changes in firing rates or synchronization are caused by pathology of the brain circuits or recording stability, especially in longitudinal AD studies. Another limitation is cell-type identification. Optical imaging can genetically label specific neuronal populations, while high-density probes generally infer neuronal identity from spike waveform properties. These indirect approaches are useful but less definitive than genetic labelling. Moreover, electrophysiological data depend heavily on spike-sorting algorithms to separate extracellular signals into putative single units. However, the spike-sorting process is still considered one of the main sources of ambiguity, especially during chronic recording experiments where changes in spike waveform properties can compromise unit identification [87]. Despite the significant improvement in the technology behind automatic spike-sorting algorithms, manual curation is still needed to increase accuracy. This increases the labour burden and creates additional sources of variability between analysts. These demands may limit accessibility and make large-scale replication difficult.

### 6.4. CMOS MEA

The CMOS MEA devices enable high-resolution measurements of electrophysiological activity in cultured neurons and acute brain slices. These devices typically incorporate tens of thousands of recording and stimulation sites. However, not all sites can be enabled simultaneously. In practice, approximately 1000 recording channels and 32 stimulation sites can be activated at one time. Given the small electrode size and the scale of the resulting datasets, spike sorting is often not routinely performed [88]. While these platforms offer great experimental control and high repeatability, their translational scope remains limited by their in vitro nature. Cultured neuronal networks lack the anatomical organisation, neuromodulatory inputs and long-range connectivity of the intact brain. On the other hand, brain slices maintain local circuitry but lose distributed network interactions. Additional practical limitations include the high cost of CMOS MEA systems (USD 200,000 to 300,000), the substantial labour required for data handling and analysis, and the difficulty of monitoring neuronal growth using conventional microscopy. Neurons cultured on CMOS MEAs may also show limited long-term viability, often surviving for less than four weeks. Moreover, in vitro Alzheimer’s disease models frequently rely on synthetic Aβ or tau exposure, which offers experimental tractability but does not fully recapitulate the chronic, multicellular progression of pathology in the intact brain. Consequently, CMOS MEA findings are best interpreted as mechanistic models of network dysfunction rather than direct biomarkers.

**Figure 2:**
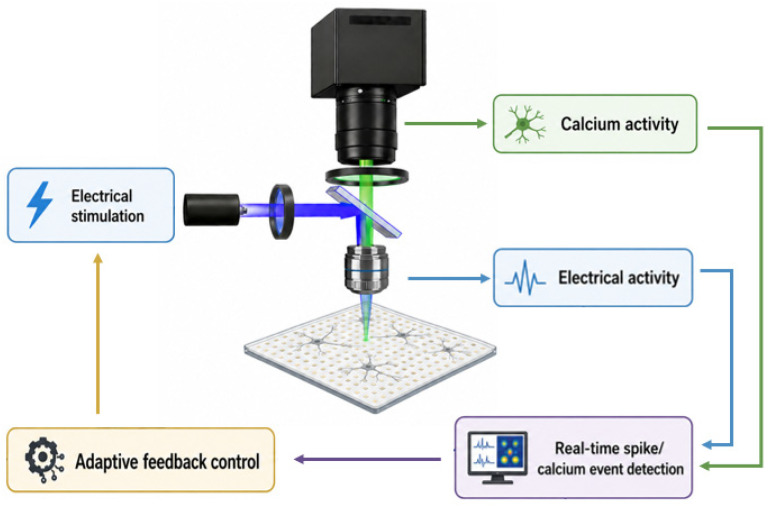
Schematic of an integrated multimodal platform combining extracellular electrophysiological recording and intracellular calcium imaging. Calcium and electrical activity are decoded in real time and used to control electrical stimulation in a closed-loop manner.

Although HD-MEA systems provide subcellular spatial resolution, they are limited to recording extracellular electrophysiological signals and cannot directly assess intracellular activity. The integration of calcium imaging and HD-MEA recording therefore provides a powerful approach for obtaining complementary functional information (Figure 2). The optical imaging approach further enables genetic targeting and three-dimensional volumetric imaging at submicron resolutions. This integrated technique will lead to improved characterization of neuronal firing patterns, and network synchronization across neuronal populations. In addition, multimodal recordings also facilitate validation of spike detection, reduce measurement ambiguity, and provide a more comprehensive description of circuit-level dynamics. Despite these advantages, multimodal integration of HD-MEA electrophysiology and calcium imaging remains relatively underexplored. To date, only one closely related study has reported partial multimodal characterisation. Quintanilla et al. combined HD-MEA electrophysiology with fluorescence imaging to achieve cellular resolution characterisation of corticospinal motor neurons [89]. In that study, the optical modality was primarily used to determine neuronal location, morphology, and maturation state on the HD-MEA platform, providing structural and cell-type context for electrophysiological signals. However, calcium signalling dynamics were not investigated. Therefore, simultaneous characterization of extracellular spiking activity and intracellular calcium signalling in large neuronal populations remains an important open challenge in future AD studies. Unlike primary rodent cultures, human induced pluripotent stem cell (iPSC)-derived neurons enable AD-related network dysfunction to be examined in a human genetic context, including patient-derived lines and isogenic models carrying defined AD-associated variants [90]. Future work could further strengthen the translational value of this hybrid system by integrating it with iPSC-derived neuronal cultures.

### 6.5. Computational Analysis and Reproducibility

Across large-scale recording modalities, the first stage of analysis usually involves translating the optical or extracellular recordings into a format of neuronal firing events for subsequent analyses. The specific pipeline differs substantially between technologies. For CMOS MEAs, the dense spatial sampling and large data volume often make simple threshold-based spike detection a practical first-line approach [88]. Although this approach is efficient for population-level analysis, different thresholding criteria can influence spike detection and may lead to variability between studies. In contrast, neural signals recorded with high-density probes require spike sorting to separate extracellular spikes, followed by quality control and often manual or semi-manual curation [87]. This process remains an important source of analytical variability. Optical imaging data introduce a different set of preprocessing challenges. Calcium and voltage-imaging movies are usually first corrected for motion artefacts using rigid or non-rigid image-registration algorithms [91,92]. For miniscope calcium imaging, constrained matrix-factorisation approaches, particularly CNMF-E, are widely used to separate neuronal signals from strong background fluorescence and infer calcium events [21]. The use of CNMF-E-based workflows has improved the robustness of calcium event extraction, but the results still depend on imaging quality and motion correction. Voltage imaging requires faster event extraction. Current approaches usually use template matching, adaptive thresholding or whitened matched filtering to improve detection of low-amplitude optical spikes [92,93]. Compared with miniscope calcium imaging, voltage-imaging analysis often involves greater manual intervention in ROI selection and spike detection, which may affect reproducibility across datasets and laboratories. Once neuronal events have been extracted, population-level activity can be analysed at several levels. Dimensionality reduction is commonly used to identify low-dimensional activity patterns within high-dimensional neural recordings [94,95]. Network analysis can then quantify synchrony, functional connectivity and hub structure across neuronal populations [96,97]. Machine-learning approaches provide a further layer of analysis by decoding behavioural state or identifying disease-related circuit signatures [98].

## 7. Conclusions

Large-scale neuronal circuit dysfunction is increasingly recognised as a key feature of AD pathology. Although traditional studies have primarily focused on molecular abnormalities and single-neuron characteristics, accumulating evidence indicates that cognitive decline is closely associated with disruptions in network-level signalling and population-wide neural integration. Earlier experimental approaches, including two-photon imaging and low-density electrode recordings, have inherent technical constraints. This review highlights recent advances in large-scale neuronal recording technologies, including miniscope imaging, optical voltage imaging, Neuropixels electrophysiology, and high-density microelectrode arrays, and discusses their application to the study of circuit dysfunction in AD (Table 1). These approaches enable high-resolution recording of distributed neural activity across in vivo and in vitro systems, thereby alleviating sampling bias arising from incomplete population sampling. Additionally, they can help identify and validate candidate functional readouts of AD-related circuit dysfunction in preclinical systems. Such readouts may also support the development of therapeutic strategies aimed at stabilising circuit function, rather than targeting molecular or cellular pathology alone. However, further work is required before these measures can be translated into clinically usable biomarkers.

Together, these studies indicate that AD-related circuit dysfunction cannot be reduced to a simple state of neuronal hyperactivity or hypoactivity. Across models, Aβ and tau pathology can be associated with increased, reduced or apparently preserved activity, yet these patterns often converge on the same functional consequence, namely a loss of coordinated and reliable information processing. The critical deficit in AD may therefore lie less in the overall level of neural activity than in how activity is organised across cells, circuits and behavioural states. Average firing rate or calcium-event frequency alone is therefore unlikely to provide a robust biomarker of disease progression. Measures that capture the precision, timing, synchronisation and connectivity of neural activity are likely to be more informative, as they more directly reflect the breakdown of circuit computation that contributes to cognitive decline.

Translational utility of these techniques depends more on identification of circuit-level features that could be translated into human context rather than use of the recording systems themselves in clinical settings. Although most of the biomarkers identified using animals, such as neural network calcium dynamics and axonal voltage propagation, cannot be measured in clinical settings, the overall dysfunction they illustrate does have clinical significance. Disrupted excitability and impaired temporal coordination may be reflected in EEG or MEG measures of spectral slowing, altered oscillatory coupling, epileptiform activity and functional connectivity [99,100]. Impaired communication between hippocampal, cortical and thalamic networks may also correspond to fMRI-based changes in resting-state connectivity, particularly within memory-related and default-mode networks [101,102]. In this sense, rodent large-scale recordings should be considered tools for discovering mechanisms that help to understand human lower-resolution recordings, but not direct biomarkers for diagnosis purposes. If validated, these non-invasive measures may be better suited for repeated assessments and longitudinal monitoring than CSF-based Aβ and tau biomarkers, which require lumbar puncture for sample collection. Finally, these functional measures in animals may facilitate drug development by providing dynamic readouts of circuit dysfunction, enabling evaluation of therapeutic efficacy and identification of treatments that restore network function. Among all measures, criticality-based measures may be particularly useful in this translational context, because they describe large-scale network dynamics and can, in principle, be extracted from lower spatial resolution recordings such as EEG [103]. Rather than depending on single-neuron resolution, this approach determines whether the neural system operates close to an optimal balance between order and disorder [39]. This balance is thought to support flexibility in information processing. Loss of this balance may therefore provide a bridge between animal-derived circuit dysfunction and clinically accessible neurophysiological signals.

Currently, the main obstacle lies in the disparity between single neuron recordings from animal experiments and the macroscopic measurements used in clinical studies. Future developments in source localization and multimodal fusion may help extract more spatially and biologically informative features from non-invasive recordings. Additionally, deep learning techniques may further increase the efficacy of EEG resolution by better reconstructing sources and uncovering hidden spatiotemporal patterns [104]. However, these computational approaches will require rigorous validation against molecular biomarkers, cognitive outcomes, and mechanistic animal data.

## Figures and Tables

**Table 1:** Summary of large-scale neural recording techniques and experimental disease models in Alzheimer’s disease studies.

Techniques	Disease Model	Year/Reference	Main Findings
Miniscope	5xFAD (in vivo)	[29], 2023	Reduced CA1 calcium activity during immobility; degraded spatial coding before overt memory impairment.
Sampling rate: 30 FPS	5xFAD (in vivo)	[30], 2025	Attenuated hippocampal calcium responses during fear conditioning, with reduced stratum pyramidale-alveus synchronisation and population consistency.
	APP/PS1 (in vivo)	[32], 2022	State-dependent hippocampal dysregulation: increased activity during exploration and NREM sleep but reduced activity during quiet rest and REM sleep.
Spatial resolution: 2–5 μm	APP/PS1 (in vivo)	[33], 2023	Tonic nucleus reuniens stimulation reduced epileptic spikes, preserved nRE-CA1 connectivity, and improved working memory and short-term plasticity.
	rTg4510 (in vivo)	[34], 2023	Tau pathology impaired visually evoked cortical ensemble responses and theta oscillations despite elevated spontaneous activity.
	3xTg-AD (in vivo)	[36], 2022	CA1 neurons were hyperactive, but spatial information and coherence were reduced, with deficits worsening with age.
	αCaMKII+/− (in vivo)	[38], 2022	Dentate gyrus populations showed impaired encoding of navigational variables, linking synaptic plasticity loss to spatial-coding deficits.
	Scopolamine (in vivo)	[40], 2026	Novel-object engagement shifted CA1 networks toward critical dynamics; scopolamine disrupted this adaptive criticality tuning.
Voltage imaging	5xFAD (ex vivo)	[46], 2024	5xFAD cortical slices showed frequency-dependent deficits in evoked signal propagation, strongest during gamma-band stimulation.
Sampling rate: 300–1000 FPS	5xFAD (in vivo)	[48], 2022	Plaque-associated axonal spheroids impaired antidromic action-potential conduction, increasing stimulation thresholds and reducing somatic responses.
	APP/PS1 (ex vivo)	[49], 2013	APP/PS1 hippocampal slices showed dentate-gyrus hyperexcitability and weakened inhibitory gating.
Spatial resolution: 2–20 μm	APP/PS1 (ex vivo)	[51], 2022	Cortical circuits showed biphasic dysfunction: early hyperexcitability before plaques and later reduced reliability and propagation after plaque accumulation.
	APP/PS1 (ex vivo)	[53], 2016	Barrel cortex showed reduced response fidelity, abnormal amplitude fluctuations, and reduced spontaneous synchrony, while evoked synchrony was largely preserved.
High-density probes	APP/PS1 (in vivo)	[58], 2025	Pre-plaque deep-layer PV fast-spiking interneurons showed reduced spiking and visual tuning, while superficial layers were relatively spared.
Sampling rate: 10,000–20,000 Hz	APP/PS1 (in vivo)	[60], 2020	Frontal pyramidal firing decreased while theta/beta LFP power and pathological phase coupling increased; levetiracetam partly normalized activity.
	TgF344-AD (in vivo)	[64], 2026	Cognitively resilient AD rats recruited fewer neurons but formed more robust stimulus representations despite preserved mean population firing.
Spatial resolution: 15–20 μm	APP/PS1- rTg4510 (in vivo)	[65], 2025	Tau selectively disrupted pyramidal burst firing and temporal coding while largely preserving single-spike activity.
	APP knockout (in vivo)	[66], 2025	APP loss reduced excitatory firing, hippocampal phase locking, coactivity, and state transitions through NMDA-receptor dysfunction.
	APP NL-G-F (in vivo)	[68], 2024	Oligodendrocyte-derived Aβ contributed to early neuronal hyperactivity; selective reduction restored circuit activity toward normal patterns.
CMOS MEA	Aβ pharmacological (in vitro)	[73], 2017	Aβ42 caused spatially heterogeneous hyperactivity and hypoactivity and disrupted burst synchrony, timing, and propagation; treatments partly rescued activity.
Sampling rate: 10,000–20,000 Hz	Aβ pharmacological (in vitro)	[77], 2023	Aβ42 oligomers weakened connectivity, reduced highly connected hubs and global efficiency, and impaired network information transmission.
Spatial resolution: 7–20 μm	Tau exposure (in vitro)	[80], 2025	Monomeric tau progressively reduced firing and burst frequency, prolonged inter-spike intervals, weakened synchrony, and increased silent units.
	Tau seeding (in vitro)	[81], 2025	Disease-relevant tau seeding triggered early hyperexcitability and hypersynchrony with rapid axonal degeneration before major neuronal loss.

## Data Availability

No new data were created or analysed in this study.
